# Enhancing primary healthcare in under-resourced communities with mobile health clinics

**DOI:** 10.1097/NSG.0000000000000116

**Published:** 2024-12-20

**Authors:** Marjory David, Marie Lourdes Charles

**Affiliations:** At Pace University in New York, N.Y., **Marjory David** is an adjunct professor and **Marie Charles** is an associate professor.

**Keywords:** Haiti, health equity, low-resource community, mobile health clinic, primary healthcare, public health nurses

## Abstract

Mobile health clinics (MHCs) have the potential to enhance primary care in low-resource communities. This article describes an initiative spearheaded by public health nurses that utilized MHCs to provide affordable, equitable, and culturally appropriate primary healthcare services to a rural community in Haiti.

Mobile health clinics (MHCs) are important in filling the gaps by providing equitable care. The use of MHCs to improve patient outcomes in low-resource and rural communities has been supported by the World Health Organization (WHO).^1^ MHCs are also endorsed by the United Nations, falling under the Sustainable Development Goals (SDGs) target number 3.8, which aims to achieve “access to quality essential healthcare services and access to safe, effective, quality and affordable essential medicines and vaccines for all.”^2^-^4^

MHCs deliver care to patients in areas with limited medical infrastructure, providers, and resources.^5^ Additionally, they link patients to local social services. MHCs facilitate care for those who do not have the time and resources to travel to traditional sites or healthcare centers.^5^ Public health nurses (PHNs) can partner with other health professionals to deliver primary care through MHCs.

PHNs work with communities and populations to foster primary prevention and health promotion using nursing, social, and public health principles.^6^

PHNs advocate, partner with communities, provide health education, and address political and social reform.^7^ As such, they are particularly suited for organizing interventions for vulnerable populations.^7^ Traditionally, primary care (PC) professionals provide healthcare throughout the lifespan.^8^ However, this is challenging in some low-resource communities because of poor access to services, increased prevalence of infectious and chronic diseases, and substandard environmental and living conditions.

PHNs can facilitate the provision of primary care through collaborations, which are effective in disease management for vulnerable populations. An important part of PHNs' responsibilities is to orchestrate cost-effective and sustainable programs.

Specialized skills such as analytic assessments allow PHNs to collaborate with individuals/communities and tailor available resources accordingly. The goal is to empower residents to meet their own healthcare needs.

This article describes an initiative involving MHCs to provide healthcare in a rural community in Haiti. The initiative exemplifies the ability of grassroots organizations led by PHNs to enhance access to healthcare.

## Background

Haiti has 1,007 health facilities, which do not cover services for the entire country. At least 122 (21%) of the 571 communal sections do not have any health facilities.^9^ Furthermore, access is difficult due to road conditions, particularly in rural areas. One in four Haitians travel over an hour to reach a health center. This proportion reaches 44% of the population in rural areas and more than half in the areas of Sud-Est and Grand'Anse.^9^ Funding for the Haitian health system relies mainly on citizens and international aid.^9^ Therefore, many Haitians in rural regions rely on MHCs as their only source of medical care.

The WHO and United Nations SDG guidelines served as the foundation for designing the Association Des Ouanaminthais (ADO) interventions.^2^,^3^ These interventions have been relevant to many low-resource communities in Haiti, which has a history of poor health outcomes and a life expectancy at birth of 64.1 years.^10^

The mortality for children in Haiti under 5 years of age is 58.6 deaths per 1,000 live births.^11^ Additionally, Haiti has a severe healthcare worker shortage, with only 10 health professionals per 10,000 inhabitants.^12^ Public health spending is among the lowest in the world. Furthermore, the earthquake in 2010 destroyed over 50 medical facilities, further dismantling the infrastructure. These challenges are especially taxing to rural communities such as Ouanaminthe.

**Table:** Implementation

Implementation mapping phases	Corresponding ADO activities
1. Identify available resources in context to the health problem, population, and causes.	Phase I was initiated in September 2014, when PHNs in Ouanaminthe, at the request of ADO, collaborated with the local Ministry of Health and stakeholders to perform a healthcare needs assessment. Data on population demographics, endemic diseases, chronic diseases, and patient perspectives were collected. The needs identified included health education, disease prevention, diagnostics, and treatment.
2. Create objectives to impact behavior and environmental causes.	Due to the nature of the intervention, the behavioral and environmental objectives were not indicated. The program was to provide primary care to patients.
3. Identify theory for behavior change.	The program was not intended to change behaviors.
4. Design an intervention plan.	ADO leadership recruited a team of facilitators and educators, including those from the US and Canada who participated in several preparatory meetings to discuss training topics and approaches. Fundraising was undertaken, and monies were utilized to pay for medications, supplies, fees for the venue, and travel expenses for Haiti-based volunteers. Additionally, national and international medical outreach programs furnished some medications and supplies. Performance expectations and tasks were delineated according to the roles.
5. Implementation of the program	Implementation occurred from January through August 2015 and was orchestrated by the ADO leadership and international and local providers/PHNs. Publicity and marketing for the event were coordinated at least 3 months before its launch. Training for local healthcare practitioners was delivered on August 11, 2015, 2 days before the MHC. The support team, which consisted of technological experts, clerical staff, and security staff, participated in operation logistics. Patients were triaged through a preregistration process the day before and given a bracelet. The MHC was launched on August 13, 2015.
6. Evaluate outcomes and sustainability.	Evaluation of the project started in August 2015, on the day of the intervention, and consisted of (a) preliminary appraisal of the activities of the clinic; (b) performance appraisal of the team and providers; (c) informal patient interviews; and (d) sustainability and maintenance factors. At the end of December 2015, decisions to continue the yearly clinic were made in conjunction with ADO, the local Ministry of Health, and other stakeholders.

Ouanaminthe is in the Northeast region of Haiti, with a population of approximately 110,000.^13^ Most residents are farmers with a low literacy level. They depend on international aid from nonprofit organizations to supplement healthcare.

ADO is a nonprofit organization whose vision is to build a bridge between the Haitian diaspora mainly from the US and Canada and the people of Ouanaminthe by providing health education, stewardship, and cultural development.^14^ Diaspora refers to a community of people who live outside a shared country of origin, ancestry, or affinity but maintain group identity.^15^ Due to various relationships with the people and firsthand knowledge of their needs, the diaspora is well-suited to advocate for interventions and policies. The United States Department of State – Global Diaspora Forum acknowledged the diaspora's financial and advocacy contributions.^16^ For example, some diaspora invest in their homeland through housing and financially supporting entrepreneurship and institutions. Furthermore, they collaborate with internal stakeholders to improve conditions in their homeland.

**Figure:**
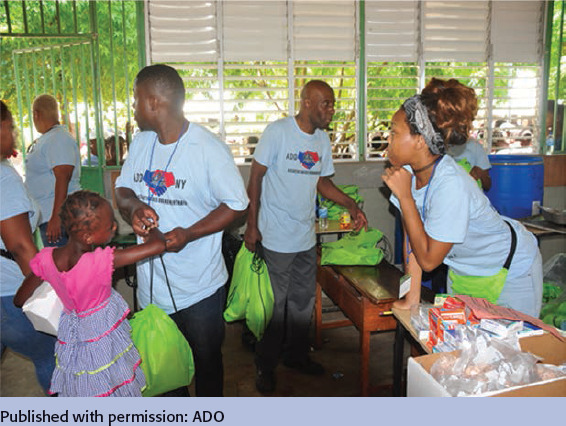
Medication and gift distribution

Anecdotal reports from partners living in Haiti regarding healthcare services in Ouanaminthe led to the initiation of a healthcare needs assessment in September 2014 in collaboration with the local Ministry of Health. The healthcare needs assessment revealed a high prevalence of hypertension, diabetes, skin, and other infections among residents. In response to these results, several local organizations collaborated with ADO to organize the first mobile clinic in 2015. These mobile clinics have been offered annually except during the pandemic.

## Purpose

The objectives of the ADO initiative were to ensure health equity through “health promotion, disease prevention, and treatment”.^4^ ADO's goals were to reduce healthcare disparities by providing Ounaminthais better access to primary healthcare services that were inexpensive, equitable, and culturally appropriate.

## Methods

This initiative consisted of four steps adapted from the implementation mapping process by Fernandez et al.^17^ Since the program was not intended to change behaviors, steps two and three were not included (see *Implementation*).

ADO PHNs used information from the needs assessment to determine clinical necessities and costs and to design and execute the first mobile clinic for this area in August 2015. Fundraising efforts included a Mother's Day Brunch and a holiday celebration at the end of the year.

The ADO team was led by Haitian PHNs with familial connections with the area and culture; therefore, they knew the region as their home. The team also included recruited and volunteer healthcare professionals such as physicians, NPs, and RNs from the US and Canada, many of whom were trained and practiced in Haiti. Additionally, they were responsible for financing personal travel and lodging. ADO paid for transportation, meals, and lodging for Haiti-based volunteers.

Preparation for the event included a campaign to inform residents of the upcoming MHC. This approach resulted in the MHC being publicized through different media formats, including blowhorn announcements. Training consisted of reviewing the intake form, plan of care, therapeutic communication, assessment skills, and use of diagnostic instruments.

The ADO team conducted the MHCs in local schools (see *Medication and gift distribution* and *A patient receiving dental care*). The means of transportation include motorcycles, sports utility vehicles, bicycles, and on foot. At times, transportation is provided for participants.

Attendees were evaluated to ensure competence. Preregistration was a crowd control measure and occurred the day before. As there were no emergencies, patients from neighboring towns and those who had never seen a physician or a nurse were given priority.

Additionally, unregistered patients were not seen until the end of the clinic day, around 1700 hours. It was difficult to estimate the number of patients not seen because unregistered patients came on a walk-in basis.

## Participants

The target population consisted of patients of all age groups and specialties, from Pediatrics to Gerontology. From 2015 to 2019, over 4,900 individuals received services. The MHC was held every August. All the patients were screened by PC physicians and/or NPs and pediatric providers (see *Patient screening*).

Several patients received services from different specialties on the same day, as referred, such as obstetrics/gynecology (OB/GYN), dental, psychiatry, and optometry.

## Results

The MHC providers found the following prominent health conditions: hypertension, elevated serum cholesterol, glucose, and A1C; gastrointestinal problems; malnutrition; tapeworms; and skin problems.

The most critical patients were connected to local health centers and hospitals for follow-up. In 2015, a patient presented with tachycardia, hypotension, and other symptoms of severe anemia and was referred for further evaluation, resulting in a blood transfusion. In 2017, another patient was screened with a PAP test, had abnormal results, and was subsequently connected to Oncology services. In 2022, a 5-day-old patient presented with no anus on assessment and was referred for colostomy. In 2023, a patient with a facial mass was referred for surgery.

Teaching about infection control and prevention was also essential in addressing skin and gastrointestinal infections. Simple techniques and interventions such as hand hygiene, boiling water, or adding a water purification tablet can impact the outcomes. Findings from the data analysis for the 2022 MHC led to the design of a referral system for patients to ensure continuity of care. However, this program's limited funding initially enrolled only 10 patients.

**Figure:**
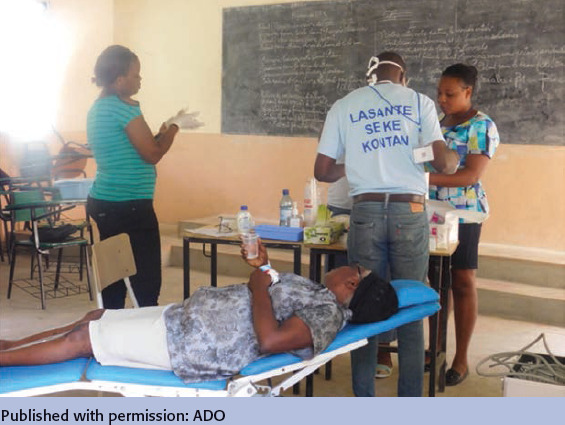
A patient receiving dental care

## Discussion

Data from chart reviews and patient interviews supported that the mobile clinics were being utilized outside their intended use of screenings, initiating preventive care, managing chronic diseases, and promoting self-efficacy. Nevertheless, the clinic was a 1-day intervention, where patients were given medications, hygiene kits, and reading glasses. Patient teaching was also conducted in individual and group sessions. Short-term indicators of success included patients verbalizing (a) the importance of diet and medication adherence, (b) understanding of water purification methods, and (c) listing signs and symptoms requiring immediate medical attention. Patients were instructed to follow up with local hospitals and clinics to obtain medications and ensure continuity of care. However, connecting patients for follow-up care was problematic because of the lack of local healthcare centers and expenses for medications and transportation to these facilities.

Evaluating if the interventions affected morbidity and mortality was beyond the scope of the MHC, as the intention was to provide primary care through a 1-day venue. Furthermore, logistics challenges and political unrest have impacted the ability to collect data from the Ministry of Health in Haiti.

Subsequent evaluation included determining the cost-effectiveness of how many patients were seen. The following ongoing goals were identified: improving the training and orientation process offered to volunteer staff and streamlining limited resources.

The following activities are also ongoing: fundraising, recruiting volunteers, creating a referral system, and forming relationships with Haiti-based partners.

**Table:** Patient screening

Year	Specialty/Patients seen	Age range	Total	Referrals
2015	OB/GYN = 20 PC = 240 PSYCH = 5 DENTAL = 192 OPTOMETRY = 221 PEDIATRICS = 343	Adults 21 to 85 years Pediatrics 3 days to 21 years	513	one patient with severe anemia
2016	OB/GYN = 30 PC = 513 PSYCH = 0 DENTAL= 187 OPTOMETRY = 210 PEDIATRICS =310	Adults 21 to 98 years Pediatrics 1 to 21 years	823	0
2017	OB/GYN = 50 PC = 617 PSYCH = 6 DENTAL = 248 OPTOMETRY = 315 PEDIATRICS = 407	Adults 21 to 90 years Pediatrics 6 months to 21 years	1,024	one patient with abnormal PAP test
2018	OB/GYN = 40 PC = 780 PSYCH = 2 DENTAL = 308 OPTOMETRY = 311 PEDIATRICS = 435	Adults 21 to 95 years Pediatrics 1 month to 21 years	1,215	0
2019	OB/GYN = 30 PC = 769 PSYCH = 2 DENTAL = 240 OPTOMETRY = 302 PEDIATRICS = 577	Adults 21 to 93 years Pediatric 5 months to 21 years	1,344	0
2020			0	
2021			0	
2022	OB/GYN = 10 PC = 408 PSYCH = 0 DENTAL = 145 OPTOMETRY = 115 PEDIATRICS = 210	Adults 21 to 94 years Pediatrics 5 days to 21 years	618	10 patients for chronic disease and one 5 day-old born with no anus
2023	OB/GYN = 10 PC = 524 PSYCH = 0 DENTAL = 226 OPTOMETRY = 310 PEDIATRICS = 439	Adults 21 to 95 years Pediatrics 6 months to 21 years	963	18 patients, including one patient with facial mass	

### Lessons learned

A concern was the number of patients using the MHC as their only source of healthcare. As patients learned of services provided at the MHC, their interest grew, and the ADO responded by expanding to a 2-day offering. Patient teaching was pivotal in disease self-management. More patient teaching sessions were added to the program.

Another challenge was patient follow-up. The ADO initiated and continues to work to refine a referral system for the most acute cases; however, limited resources and funding have impeded progress. The same site would be used for the clinic for continuity and sustainability.

ADO and Haiti-based PHNs also benefited from the project by expanding their role to promote health equity in their community.

## Conclusion

The ADO initiative illustrates how public health professionals can effectively establish an MHC to help under-resourced communities. Such programs also allow public health professionals to effect sustainable change. A proactive way to implement these services is through partnerships with grassroots organizations and community leaders.

Although the program faced challenges, including the COVID-19 pandemic, poor funding, a travel ban, and civil unrest, the ADO initiative is ongoing and expanding to ensure the provision of healthcare services throughout the year from local clinics.
